# Non-Invasive Targeted Peripheral Nerve Ablation Using 3D MR Neurography and MRI-Guided High-Intensity Focused Ultrasound (MR-HIFU): Pilot Study in a Swine Model

**DOI:** 10.1371/journal.pone.0144742

**Published:** 2015-12-14

**Authors:** Merel Huisman, Robert M. Staruch, Michelle Ladouceur-Wodzak, Maurice A. van den Bosch, Dennis K. Burns, Avneesh Chhabra, Rajiv Chopra

**Affiliations:** 1 Department of Radiology, UT Southwestern Medical Center, Dallas, TX, United States of America; 2 Department of Radiology, University Medical Center Utrecht, Utrecht, The Netherlands; 3 Department of Pathology, UT Southwestern Medical Center, Dallas, TX, United States of America; 4 Clinical Sites Research Program, Philips Research North America, Briarcliff Manor, NY, United States of America; University Hospital-Eppendorf, GERMANY

## Abstract

**Purpose:**

Ultrasound (US)-guided high intensity focused ultrasound (HIFU) has been proposed for noninvasive treatment of neuropathic pain and has been investigated in in-vivo studies. However, ultrasound has important limitations regarding treatment guidance and temperature monitoring. Magnetic resonance (MR)-imaging guidance may overcome these limitations and MR-guided HIFU (MR-HIFU) has been used successfully for other clinical indications. The primary purpose of this study was to evaluate the feasibility of utilizing 3D MR neurography to identify and guide ablation of peripheral nerves using a clinical MR-HIFU system.

**Methods:**

Volumetric MR-HIFU was used to induce lesions in the peripheral nerves of the lower limbs in three pigs. Diffusion-prep MR neurography and T1-weighted images were utilized to identify the target, plan treatment and immediate post-treatment evaluation. For each treatment, one 8 or 12 mm diameter treatment cell was used (sonication duration 20 s and 36 s, power 160–300 W). Peripheral nerves were extracted < 3 hours after treatment. Ablation dimensions were calculated from thermal maps, post-contrast MRI and macroscopy. Histological analysis included standard H&E staining, Masson’s trichrome and toluidine blue staining.

**Results:**

All targeted peripheral nerves were identifiable on MR neurography and T1-weighted images and could be accurately ablated with a single exposure of focused ultrasound, with peak temperatures of 60.3 to 85.7°C. The lesion dimensions as measured on MR neurography were similar to the lesion dimensions as measured on CE-T1, thermal dose maps, and macroscopy. Histology indicated major hyperacute peripheral nerve damage, mostly confined to the location targeted for ablation.

**Conclusion:**

Our preliminary results indicate that targeted peripheral nerve ablation is feasible with MR-HIFU. Diffusion-prep 3D MR neurography has potential for guiding therapy procedures where either nerve targeting or avoidance is desired, and may also have potential for post-treatment verification of thermal lesions without contrast injection.

## Introduction

Chronic neuropathic pain and cancer pain are potentially debilitating conditions that seriously reduce the quality of life [1, 2]. In cases where the symptoms are not responsive to conservative measures, temporary or permanent disruption of neural pathways may be necessary to achieve pain relief, which can be performed surgically or by means of minimally-invasive procedures [3–5]. Minimally-invasive treatment options include image-guided injection of a long-lasting local anesthetic (nerve block), or radiofrequency thermal ablation of the affected nerve using guidance with ultrasound (US), fluoroscopy, computed tomography (CT), or magnetic resonance imaging (MRI) [6–10]. Fluoroscopy guidance is widely available, but the neurovascular area can only be targeted using bony landmarks that lends the technique to potential nerve injury or missing the intended target altogether [6, 11]. CT guidance has similar limitations in inexperienced hands and involves undesirable exposure to ionizing radiation for both the patient and the clinician[6]. Ultrasound guidance is suitable for superficial targets, but is ineffective at identifying deeply situated nerves[12]. MRI provides inherently high spatial resolution and superb soft tissue contrast that allows identification of deeply situated nerves [6]. In particular, MR neurography uses suppression of signal from fat, blood vessels, and surrounding muscle, to directly visualize and map the course of deep pelvic nerve targets with exceptional contrast [13] Therefore, MR neurography guidance could be advantageous in interventions where visualization of peripheral nerves is important, i.e. to target the nerves accurately and/or to prevent neurologic damage during bulk soft tissue ablation. Regardless of the image guidance technique, minimally-invasive nerve block and neurolysis require technical skills and insertion of either an injection needle or a radiofrequency probe, potentially increasing the risk of complications (e.g. infections or direct nerve damage)(6).

High-intensity focused ultrasound (HIFU) is a completely non-invasive modality capable of producing millimeter-sized thermal lesions, several centimeters beneath intact skin [14]. Clinical systems incorporating a HIFU transducer into an MR imaging system (MR-HIFU) enable treatment planning and precise ablation volume control using dynamic MR temperature mapping [15]. MR-HIFU is being investigated for use in soft-tissue ablations [16–18], and has been proven to be safe and effective for the palliation of metastatic bone pain through thermal ablation of the periosteal nerves [19]. Denervation with MR-HIFU has also been applied to the treatment of chronic arthritic pain in the knee [20] and the facet joint [21, 22]. Possibly, denervation with MR-HIFU could be extended to the treatment of peripheral neuropathic pain, offering a non-invasive alternative to neurolysis or neurectomy. Preclinical studies using US-guided HIFU have shown the capability of HIFU to induce a reversible partial conduction block or irreversible conduction block [23–26], and earlier work identified a relationship between thermal dose and the extent of changes in peripheral nerve histology and function [27]. The feasibility of using MR-HIFU for intercostal neurolysis was recently investigated in a swine model, although localization of the nerves relied on the visualization of the ribs on MRI, as no attempts were made to visualize the nerves using MR imaging [28]. Monteith et al [29] demonstrated temperature elevations of the trigeminal nerve with MR-HIFU using a gradient echo-sequence.

In the present study, the first experience with using 3D MR neurography to guide MR-HIFU for targeted nerve ablation in a swine model is presented. The primary purpose of the study was to evaluate the feasibility of utilizing 3D MR neurography to identify and guide ablation of peripheral nerves using a clinical MR-HIFU system. The extent of ablation and neuronal damage was evaluated using a combination of MR imaging, MR temperature data, and histologic data.

## Materials and Methods

### Animals

These experiments were approved by the local Institutional Animal Care and Use Committee at UT Southwestern Medical Center (protocol number APN 2013–0090). This study conforms to the National Institutes of Health’s PHS Policy on the Humane Care and Use of Laboratory Animals, and with the Animal Welfare Act and Guidelines enforced by the USDA.

Three female pigs (Yorkshire and Hampshire breeds) weighing 50–75 kg (Change of Pace, Aubrey, TX) were anesthetized with tiletamine and zolazepam (Telazol®, 5 mg/kg) and atropine (0.02–0.05 mg/kg) intramuscularly. The animals were masked with 3–5% isoflurane and then intubated with an 8–9 Fr cuffed endotracheal tube. General anesthesia was maintained with 2.0–3.5% isoflurane delivered via mechanical ventilation (10–15 ml/kg, 15–20 breaths per minute) using a large animal ventilator (Hallowell EMC Multiflow 2002, Pittsfield, MA). A 22-gauge angiocatheter was placed in the marginal ear vein to allow the delivery of intravenous drugs. Both hind legs were carefully shaved, chemically depilated (Veet®), cleaned and inspected to prevent the presence of air or skin defects that might interfere with ultrasound beam propagation. To ensure the appropriate depth of anesthesia, heart rate and oxygen saturation were monitored using an MR-compatible physiological monitor (Nonin 8600V with 30’ fiber-optic cable, Plymouth, MN). A circulating water blanket (Gaymar TP-500, Orchard Park, NY) was used to maintain core body temperature, and rectal and intramuscular temperatures were monitored using MR-compatible fiber optic probes (Neoptix Inc, Quebec, Canada). Immediately following the experiment, anesthetized animals were humanely euthanized via overdose of injectable anesthetic (Euthansol®, 100mg/kg, IV).

### MR-HIFU System and Experimental Set-Up

All experiments were performed using a clinical MR-HIFU system (Sonalleve V2, Philips Medical Systems, Vantaa, Finland), integrated into a 3T MR scanner (Ingenia, Philips Healthcare, Best, The Netherlands). The MR-HIFU system is clinically approved (not in all jurisdictions) for the treatment of uterine fibroids and for palliation of painful bone metastases. The HIFU tabletop contains a 256-element phased array focused ultrasound transducer (diameter 140 mm, operating at 1.2 or 1.4 MHz). All power values displayed to the user and quoted in the manuscript are in acoustic watts, calculated by the system using a power calibration table derived from radiation force balance measurements. The system performs an automated electrical matching routine with the subject positioned on the HIFU table prior to the first therapeutic sonication to optimize the electrical to acoustic conversion efficiency. Within the range of powers used, this value is approximately 40%. Estimations of focal intensity based on hydrophone measurements made in water at 10W suggest that for acoustic powers of 160-300W used in this study, with an attenuation of 5 Np/m, the spatial-peak temporal-average intensities would be approximately 3.7 to 6.5 kW/cm^2^.

Specific to this system is the concept of volumetric ablation [15]; in a single ultrasound exposure (sonication) the ultrasound focus is electronically steered along concentric circular paths to create ablation volumes (referred to as treatment cells) greater than the natural focal point size (approximately 1.0 x 1.0 x 7.0 mm^3^).

For targeting and sonicating the sciatic nerve, animals were placed above the ultrasound transducer on the MR-HIFU tabletop in the MR scanner in a supine position, slightly tilted towards the leg of interest. To ensure air-free acoustic coupling, and to offset the animals to an appropriate distance from the transducer, a 15 mm thick acoustically transparent gel pad was used (Aquasonic, Parker Laboratories, Fairfield, NJ). MR signal was received using the five-element coil array of the MR-HIFU system, including a two-element receive coil built into the acoustic window in the tabletop, and a three-element receive coil array placed around the dorsal surface of the animal.

### MR Imaging

**Fig 1:**
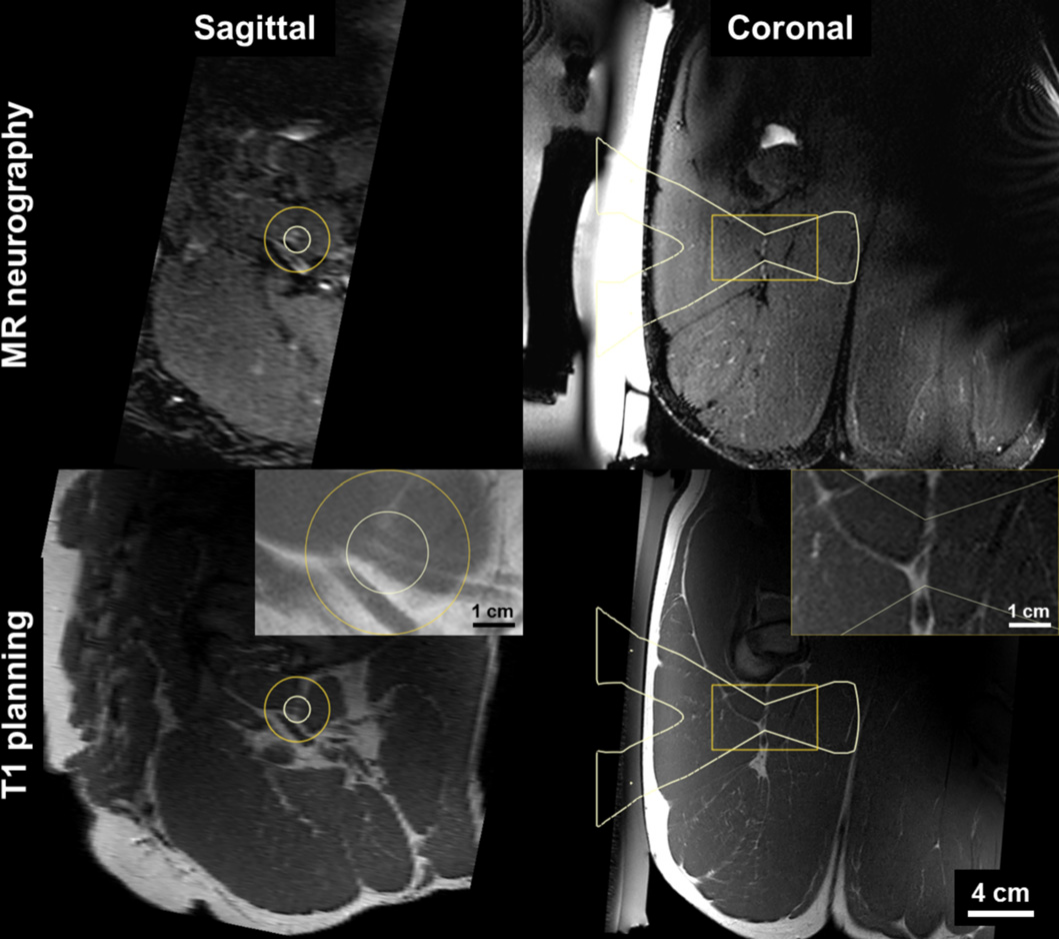
Targeting of peripheral nerve using MR-HIFU treatment planning system. The sciatic nerve is hyper-intense on MR neurography and iso-intense on T1 where dual-bundle structures are seen against fat. HIFU beam overlay (white) indicates 12 mm diameter target region.

Once the animal was positioned correctly on the MR-HIFU system, pre-treatment images were acquired for nerve identification and treatment planning using T1-weighted (T1W) and 3D isotropic MR neurography sequence. T1W images were acquired in an oblique coronal orientation to obtain high resolution visualization of the sciatic nerve bundle (Fig 1). For MR-neurography, in this study the SHINKEI (nerve-SHeath signal increased with INKed rest-tissue RARE Imaging) pulse sequence was used to acquire high-resolution 3D isotropic images [6, 13, 30]. Briefly, the sequence uses a velocity encoding pre-pulse that effectively produces low b-value diffusion weighting in all three directions resulting in suppression of signal from the flowing blood. The latter technique along with uniform fat suppression from adiabatic inversion recovery, and a long echo time suppressing T2-weighted muscle signal while preserving endoneurial fluid signal from the nerve leads to good peripheral nerve depiction. Nerves were identified on SHINKEI images as intermediate intensity uniform trajectories relative to near completely suppressed intermittent signal from the blood vessel walls (completely suppressed signal in arteries and near completely suppressed signal in veins) which appeared hyperintense to the surrounding muscle. Thick slab (15 mm) maximum intensity projection images were reconstructed in multiple oblique planes in conjunction with high resolution T1W corresponding images were used to identify the nerve tracks prior to using thin slices for positioning of the treatment cells (Fig 1). T1W images were also used to identify any obstacles to ultrasound propagation in the beam path, such as proximity to the ischium or posterior column of acetabulum.

During sonication, temperature changes were measured with the proton resonance frequency-shift (PRFS) MR-thermometry method using a multi-slice fast field echo (FFE) sequence with echo planar technique (EPI), in five image planes across the ultrasound beam and one plane along the beam axis. The peak temperature was defined as the maximum temperature in a voxel in the target zone. Immediately after the treatment, SHINKEI images were acquired in the same manner as above, and T1W images were obtained before and after contrast media administration (0.02 mmol/kg, Gadovist) to evaluate hyperacute tissue changes following ablation. For sonications in the first treated leg of the animal, contrast-enhanced T1-weighted (CE-T1) images were acquired after the last sonication was completed. Detailed imaging protocols are listed in Table 1.

**Table 1:** MRI protocol for MR-HIFU for peripheral nerve ablation.

Protocol	Sequence
Preprocedural planning	
*T1-weighted*	3D T1 FFE; axial
	TE/TR 2/3.9 ms
	FOV: 240 x 360 x 200
	Resolution: 1 x 1 x 1 mm, NSA 2
*T2-weighted*	3D TSE DRIVE (T2w), sagittal
	TE/TR 135/2001 ms, TSE factor 74, es 5.8 ms
	FOV: 250 x 270 x 200
	Resolution: 1.25 x 1.25 x 1.3 mm, NSA 1
*MR-neurography (SHINKEI)*	3D TSE SPAIR T2prep (50 ms, venc 1 cm/s)
	TE/TR 142/2500, TSE factor 60, es 4.1 ms
	FOV: 320 x 320 x 100
	Resolution: 1.09 x 1.09 x 0.9 mm, NSA 1
MR-thermometry	M2D T1 FFE, EPI factor 11
	TE/TR 16/25, flip angle 20°, water-fat shift 5 pixels, ProSet 121 Fat suppression
	FOV: 400 x 400
	Resolution: 2.083 x 2.083 x 7 mm
	Dynamic scan time 2.6 seconds (6 slices)
Postprocedural assessment	3D T1 FFE mDIXON water-only
	TR/TE1/TE2 5.1 / 1.37 / 2.5 ms, Flip 10
	FOV: 350 x 350 x 180
	Resolution: 1.25 x 1.25 x 1.25 mm, 0:59, NSA 4

### Targeted Nerve Ablation Using MR-HIFU

For each target location in the nerve, a single sonication was performed to generate the ablation. The cross-sectional diameter of the treatment cells used in this study was 8 and 12 mm, with a length of 20 and 30 mm, respectively. Standard non-feedback treatment cells were used, with fixed sonication durations of 20 seconds for 8 mm treatment cells and 36 seconds for 12 mm treatment cells. Due to the large size of the sciatic nerves, large treatment cell sizes were chosen to cover the full width of each targeted nerve. The treatment cells were positioned approximately perpendicular to the nerve. All ultrasound exposures were performed at a frequency of 1.2 MHz. Before each therapeutic sonication, one to three test sonications at 40–100 W were performed for calibration of the ultrasound focus location. In total, seven therapeutic sonications were performed in six peripheral nerves of the lower limb (sciatic nerve, n = 5; muscular branch of the sciatic nerve, n = 1). One contralateral sciatic nerve was used as an untreated control for this study. The exposure powers were varied to evaluate the degree of thermal damage as a function of temperature. Detailed experiment parameters, including targeted nerve, treatment cell sizes and acoustic power are listed in Table 2. After sonication, the skin overlying the target area was inspected visually for any skin burns.

**Table 2:** Characteristics of consecutive experiments.

Animal #	Target	Target depth (mm)	Treatment cell diameter (mm)	Acoustic Power (W)^a^	Sonication duration (s)	T max (°C)^b^	Estimated I_SPTA_ (W/cm^2^)^c^
1	SCN left	Control	Control	Control	Control	Control	Control
1	SCN right, distal	60	8	160	20	60.3	3700
1	SCN right, proximal	60	8	200	36	64.7	4600
2	SCN left	57	12	240	36	71.3	5700
2	Muscular branch of SCN left	66	12	300	36	63.3^d^	6500
2	SCN right	63	12	280	36	85.7	6300
3	SCN left	71	12	280	36	70.4	5800
3	SCN right	75	12	280	36	82.5	5500

Abbreviations: SCN, sciatic nerve; mm, millimeter, W, watt; s, second; T max, maximum temperature; °C, degrees Celsius; I_SPTA_, intensity, spatial peak temporal average.

^a^ Acoustic efficiency was approximately 40%

^b^ As determined by the Sonalleve software

^c^ Estimated from hydrophone measurement in water, derated using 5 Np/m attenuation

^d^ Relatively low Tmax was likely due to presence of a vessel within the sonication region.

### Lesion Analysis

The qualitative appearance of ablated nerve sections on pretreatment T1W and SHINKEI was compared to the corresponding post-treatment imaging. Maximal lesion dimensions were measured on CE-T1 and SHINKEI images reconstructed across and along the beam path such that the lesion diameter corresponded with the damage along the nerve, while the lesion length corresponded to the extent of muscle damage. On CE-T1, the non-perfused volume (NPV) as well as enhancing area was measured. Thermal dose maps calculated by the clinical treatment planning software were used to measure corresponding dimensions of the region that received a thermal dose of at least 240 equivalent minutes at 43°C (EM). All imaging measurements were performed using 3D-reformatted images displayed in the clinical treatment planning system (Sonalleve MR-HIFU, Philips Medical Systems, Vantaa, Finland), in consensus by two observers (R.S., 6 years of experience and M.H., 4 years of experience). Macroscopic lesions were photographed next to a ruler using a digital camera (Canon USA Inc., Lake Success, NY). The lesion diameter along the nerve was determined in the image analysis software ImageJ (NIH, Bethesda, MD), using the ruler in the image for scale. The length of the lesion was not measured macroscopically.

### Histology

Within three hours of the sonication, the targeted segments of the sciatic nerve were harvested, extending 3 cm proximal and distal to the macroscopic lesion. All nerve segments were pinned flat to dental wax to prevent recoiling and immersed in 10% buffered formaldehyde. In the first pig, the right nerve was sectioned transversely through the proximal mid-lesion and up to 2 cm proximal to this lesion to gauge the general extent of the damage. In the second and third pig, the nerve segments were sectioned transversely through the center of the lesion and at 6–7 mm intervals up to about 2 cm proximal and distal to the lesion. This was done to evaluate the spatial extent of damage to the nerve. The tissue samples were paraffin-embedded, sectioned into 5 μm slices, and stained. Hematoxylin and eosin (H&E) staining was used to observe the overall morphology and the nuclei of Schwann cells, and Masson’s trichrome was used to visualize the myelin sheaths. In addition, to allow for examination of the structural details of myelinated and unmyelinated fibers, a transverse segment of one lesion (left muscular branch of pig 2) was post-fixed in 3% buffered glutaraldehyde and epon-embedded, after which 1.5 μm semi-thin sections were obtained and stained with toluidine blue. Histology slides were reviewed by an independent neuropathologist with greater than 30 years of experience (D.B.), blinded to the exposure conditions for each section, to identify the extent of hyperacute nerve damage on bright field microscopy (Eclipse E600, Nikon, Japan).

## Results

### In-Vivo Study

**Fig 2:**
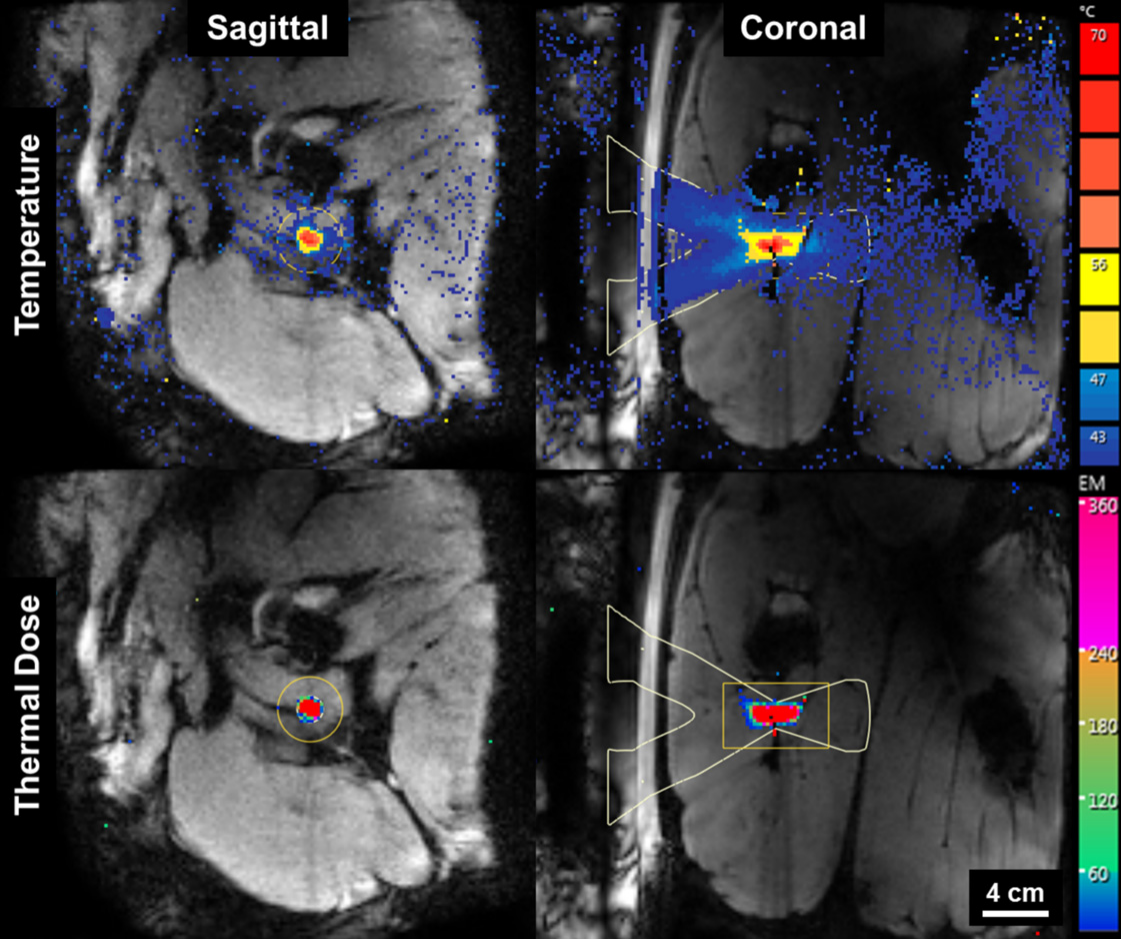
MR thermometry guidance of HIFU thermal ablation of peripheral nerves. Temperature maps (top) indicate peak temperature of 82.5°C. Thermal dose maps (bottom) predict lesion size of 12 x 29 mm.

**Fig 3:**
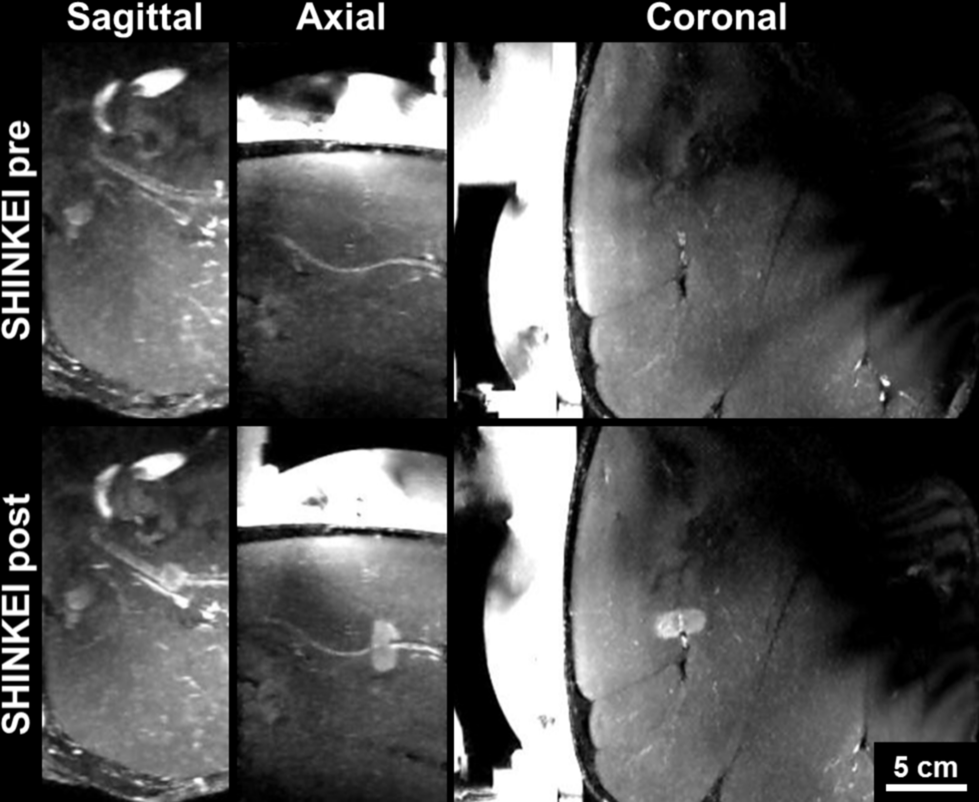
MR neurography of pig sciatic nerve before (top) and after (bottom) MR-HIFU. SHINKEI images reconstructed as thick-slab (15 mm) maximum intensity projections in sagittal, axial, and coronal orientations.

**Fig 4:**
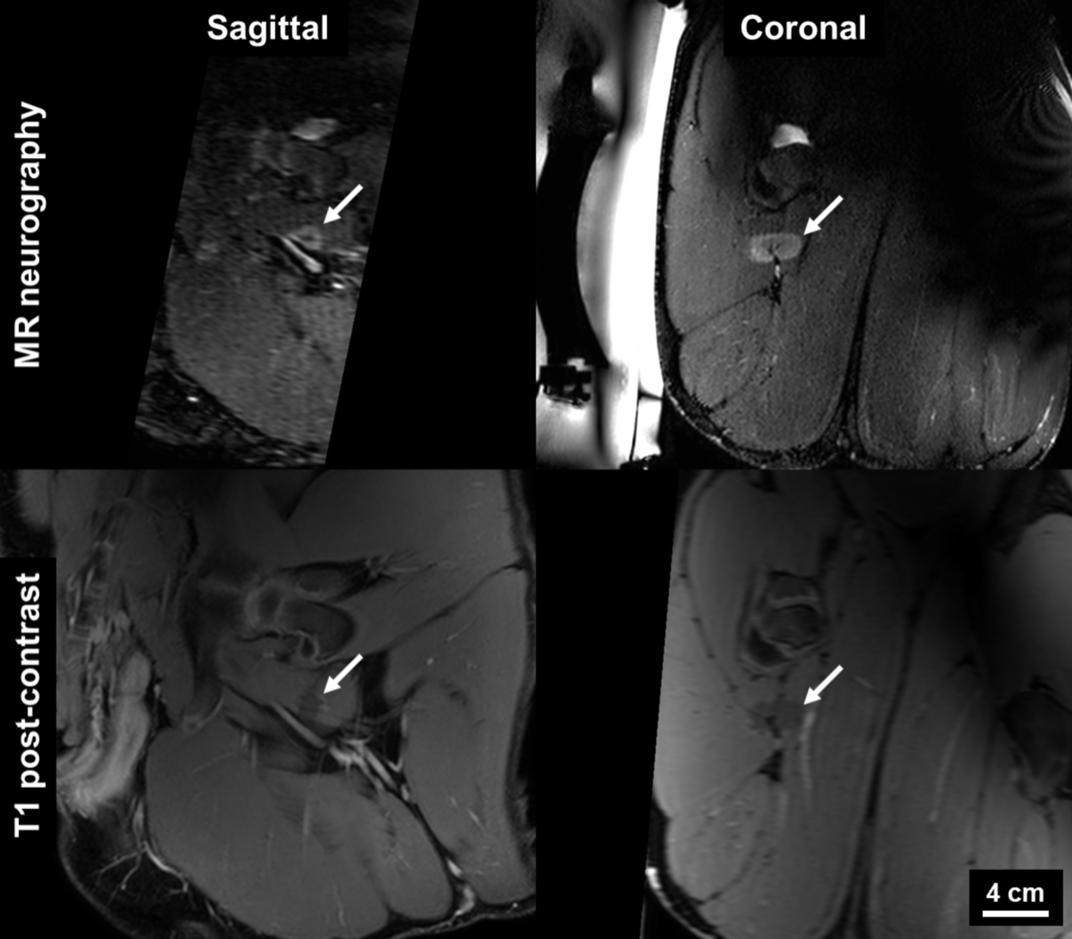
MRI evaluation of thermal lesions in peripheral nerve. Hyperintense region on 3D diffusion-prepared MR neurography (top) corresponds with non-enhancing region on T1 post-contrast image.

**Fig 5:**
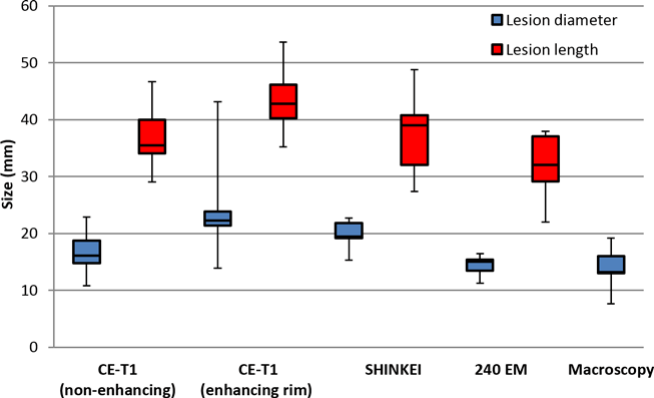
Boxplot of lesion diameter and length in 12 mm treatment cells (n = 5) as measured on imaging, thermometry and macroscopy.

The procedures were well tolerated by all animals and no skin burns occurred. In the first pig, pressure sores on the skin overlapping with the tail and the knees were observed, due to prolonged supine positioning and pressure of the pelvic coil. Image quality on T1W and SHINKEI sequences was sufficient in all animals to identify peripheral nerves as small as 3 mm in diameter. All sonications were completed successfully with a suitable acoustic window leading to the nerve. Reliable MR thermometry in targets centered on peripheral nerve was achieved in 6/7 therapeutic sonications. In these sonications, the precision of temperature measurements (standard deviation in target region before heating) was 0.66 ± 0.15°C, with an average of only 2/72 voxels in the target zone being masked due to temperature uncertainty exceeding 3°C. In the left leg of pig 3, adipose tissue surrounding the neurovascular bundle resulted in low signal intensity for the water-selective thermometry scan, with 12/72 target region voxels being masked and a temperature precision of 1.39°C in the remaining voxels. Across all therapeutic sonications, the peak target region temperature measured by MR thermometry ranged from 60.3°C to 85.7°C (Fig 2, Table 2). Thermal lesions were visible as focal hyperintense regions on post-treatment MR neurography acquired before contrast injection (Fig 3), and on CE-T1, the lesions were characterized as non-enhancing regions with rim-enhancement at the periphery (Fig 4). Gross examination during muscle dissection showed obvious coagulative necrosis on the internal surface of the biceps femoris indicative of thermal damage to the muscle. The lesion on the sciatic nerve could be identified clearly as a circular lesion with a blanched center and a hyperemic rim. In 8 mm treatment cells, the lesions did not cover the full width of the sciatic nerve. In 12 mm treatment cells, lesions could be identified on the center of the nerve covering the full width of the nerve, except for the left sciatic nerve of pig 3 where the sonication was offset slightly towards the laterodorsal side of the nerve. Overall, the lesion dimensions as measured on MR neurography were similar to the lesion dimensions as measured on CE-T1, thermal dose maps, and macroscopy (Fig 5).

### Histologic Analysis

**Fig 6:**
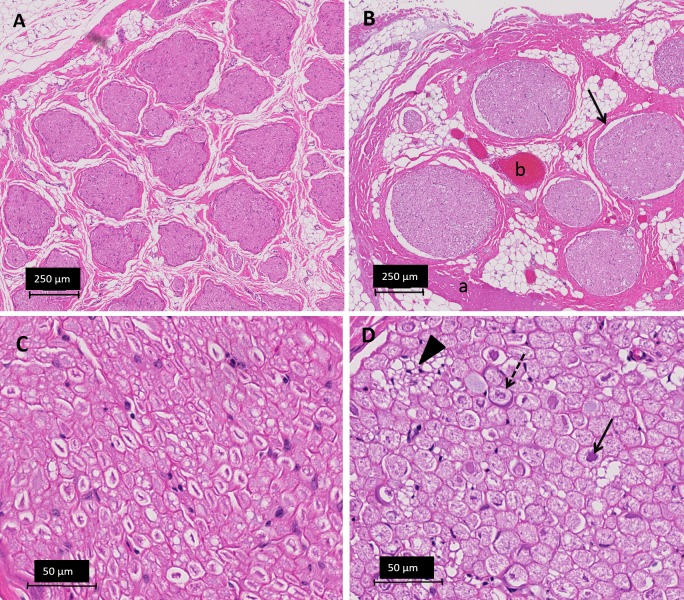
Light microscopy images of nerves, H&E staining. **A** Untreated peripheral nerve at 5x magnification. **B** Treated peripheral nerve (6 mm distal to center of ablation) at 5x magnification showing epineurial collagen homogenization (a), vascular congestion (b), widening of the subperineural space (arrow). **C** Untreated peripheral nerve 40X detail of axons and Schwann cells. **D** Treated peripheral nerve 40x detail showing regressive nuclear changes karyolysis (dashed arrow) and pyknosis (arrow) and vacuolization (arrowhead).

**Fig 7:**
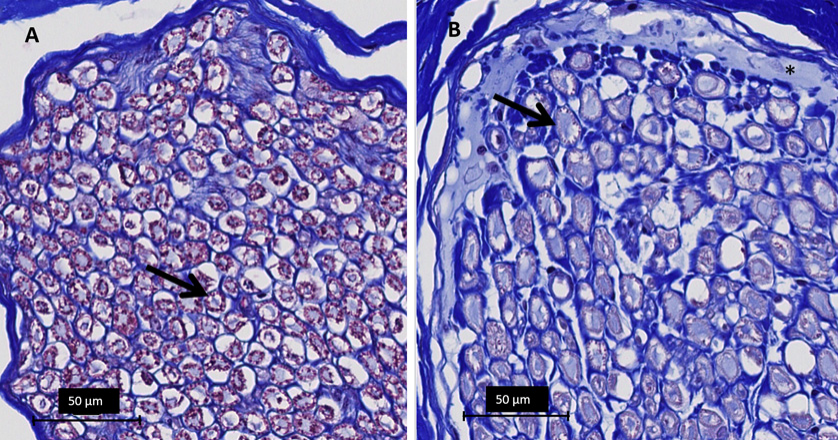
Light microscopy images of peripheral nerves at 40x magnification, Masson’s trichrome staining. **A** Normal appearance of myelin on Masson’s trichrome; myelin sheaths stain purple-red (black arrow). **B** Section of treated peripheral nerve at the center of the lesion showing absence of purple-red staining (arrow) indicating acute damage to the myelin sheaths. Asterisk (*) indicates subperineural edema.

**Fig 8:**
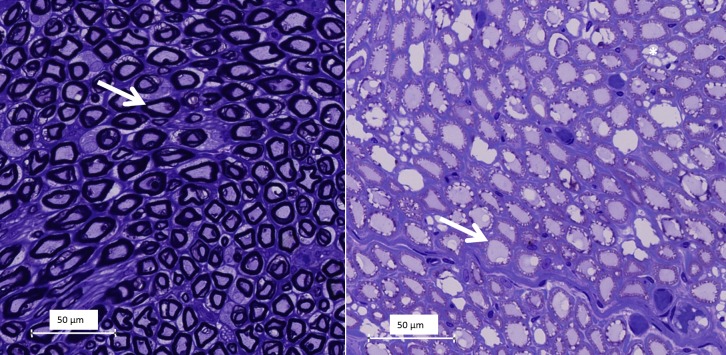
Light microscopy images at 40x magnification of semi-thin (1.5 μm) toluidine blue stained sections A Normal appearance of myelinated (arrow) nerve fibers in a cross section. B Pallor and irregularity of myelin (arrow) and vacuolization (a) in a HIFU-treated nerve segment.

Transversal sections of the lesions stained with H&E, showed vascular congestion, homogenization of epineurial collagen, subperineural edema, and pyknosis of Schwann cell nuclei (Fig 6). Transverse sections of the lesion stained with Masson’s trichrome revealed abnormally pale staining of myelin sheaths, likely indicating hyperacute myelin damage (Fig 7). On transverse sections stained with toluidine blue, the ablation caused loss of staining properties of the myelin sheaths consistent with the changes noted on the sections stained with Masson’s trichrome (Fig 8). Vacuolization was also observed on the transverse sections, consistent with swollen unmyelinated axons or Schwann cell processes (Figs 6 and 8). Analysis of the sectioned nerve samples indicated that the major damage was mostly confined to the location targeted for ablation; less extensive changes were observed at 6–7 mm away from the lesion center. At 12–14 mm away from the center, the nerve generally appeared normal (Table 3).

**Table 3:** Histological analysis of treated peripheral nerve segments.

Animal #	Proximal nerve		Lesion^a^	Distal nerve	
Distance from lesion	*+12–14 mm*	*+6–7 mm*	*0 mm*	*-6-7 mm*	*-12-14 mm*
1 SCN right,	NA	NA	No evident	NA	NA
distal			changes		
1 SCN right, proximal	Nuclear changes^a^	NA	Vascular congestion	NA	NA
			Nuclear changes		
			Widening perineural space		
2 SCN left	Normal	Widening perineural space	Vascular congestion	Normal	Normal
			Collagen damage		
			Nuclear changes		
			Myelin damage		
			Widening perineural space		
2 SCN left	Normal	Widening perineural space	Vascular	Vascular	NA
(muscular branch)			congestion	congestion	
			Collagen damage		
			Nuclear changes		
			Myelin damage		
			Widening perineural space		
			Perineural edema		
2 SCN right	Normal	Normal	Vascular congestion	Normal	Normal
			Collagen damage		
			Nuclear changes		
			Myelin damage		
			Widening perineural space		
3 SCN left	Normal	Normal	Vascular congestion, hemorrhage	Vascular congestion	Widening perineural space
			Collagen damage	Nuclear changes	Perineural edema
			Nuclear changes	Widening perineural space	
			Widening perineural space	Perineural edema	
			Perineural edema		
3 SCN right		Normal	Vascular congestion	Widening perineural space	Widening perineural space
			Collagen damage		
			Nuclear changes		
			Myelin damage		
			Widening perineural space		
			Perineural edema		

Abbreviations: SCN, sciatic nerve; NA, not available

^a^ Based on 3 cross-sections though lesion center and at 6–7 mm proximal and distal from the lesion center (lesion borders)

## Discussion

In this pilot study, the feasibility of utilizing 3D MR neurography to identify and guide ablation of peripheral nerves using a clinical MR-HIFU system is successfully investigated in a porcine model. The results of this study show that peripheral nerves identified with 3D MR neurography can be targeted and accurately ablated with a single exposure of focused ultrasound (20–36 s), since in almost all experiments, the center of the nerve was ablated. Furthermore, the MR measurements of lesion dimensions matched the dimensions observed upon surgical dissection. The temperature mapping by PRFS-thermometry appeared accurate in the targeted tissue except in case when adipose tissue was present reducing the signal due to fat suppression. On histology, nerve damage was localized to the regions of exposure with a rapid transition to normal nerve within a few millimeters, consistent with findings that have been described in the other ablated soft tissues [31].

The results of this study are in line with the work by Foley et al [24], who identified similar hyperacute histological changes in sciatic nerves of rats (i.e. myelin disruption and axon swelling) following their highest US-guided HIFU exposures. They are also in agreement with the recent work of Kaye et al [32], nerve targeting was achieved by identifying a target location with respect to adjacent fat and bony anatomy on T2-weighted fast spin-echo images, as opposed to direct visualization with background-suppressed MR neurography. One benefit of direct visualization of nerves compared to the use of anatomical landmarks was the ability to target smaller areas for ablation (approximately 0.5–1.1cm^2^ in our study vs. 4–5 cm^2^ in Kaye et al [32]). Taken in combination, the two studies demonstrate that both clinical MR-HIFU systems which were initially developed for the treatment of uterine fibroids, are capable of accurately targeting peripheral nerve tissue. In this study, some variability in peak temperatures was observed, which could only partially be explained by differences in acoustic parameters. It is well known that differences in physical (e.g. refractions of energy through tissue layers) as well as physiological factors (e.g. vascularization) all contribute to the variability of temperatures achieved in tissue for a given set of acoustic parameters, as was also observed by others [33].

The MR-neurography sequence used in this study (SHINKEI) provided treatment guidance as well as estimates of the thermal damage comparable to CE-T1 and macroscopy, without the use of a contrast agent. The latter is a notable consideration showing the potential of the non-contrast-enhanced SHINKEI sequence to monitor acute thermal damage during or directly following the treatment, with higher contrast than what is typically attainable with conventional sequences (i.e. T1, T2) [34]. In addition, MR-neurography in general may offer the capacity to enhance treatment safety of clinical MR-HIFU treatments by identifying nerves that should be avoided (e.g. the neurovascular bundle in the treatment of prostate cancer). Although in this study the SHINKEI sequence was used since it was available on the Philips MRI platform the study was performed on, MR neurography can be performed on machines from all magnet vendors. Preferably, a field strength of 3T is employed due to higher signal-to-noise ratio (SNR) and relatively fast 3D acquisition. MR-neurography employs 2D and 3D imaging. 2D imaging can be obtained using fat suppressed fluid sensitive imaging, which includes T2 SPAIR (spectral adiabatic inversion recovery; Siemens Healthcare, Philips Healthcare) and T2 Dixon (Siemens Healthcare, Philips Healthcare, GE Healthcare). The general parameters include: TR: 3800ms, TE: 60-65ms, ETL: 15, Sl: 3-4mm, in plane resolution: 0.4mm. The 3D imaging includes SHINKEI sequence as described in this paper, or 3D STIR SPACE (Siemens Healthcare) or fat suppressed CUBE (GE Healthcare). The general parameters include TR: 2000ms, TE: 76-79ms, ETL: 60, Sl: 3-4mm, in plane resolution: 0.4mm, voxel 1.2–1.5 mm isotropic.

This study has several limitations that deserve acknowledgment. First, imperative to design of this pilot study, the sample size is small and therefore the data do not allow for a valid statistical analysis of the relationship between exposure parameters and outcomes. Second, this pilot study did not include follow-up as a survival study at this stage was deemed inappropriate from an animal care point-of-view. As such, only hyperacute changes could be assessed with histology and no functional assessment was done. The thermal nerve damage observed on histology indicates damage involving both the endoneurial compartment and surrounding epineurial connective tissue, and similar changes in histology have led to acute nerve block in the study of Foley et al[24]. Presumably, the nerve damage induced in this study would have affected nerve function as well, although follow-up functional studies (e.g. nerve conduction studies) would be needed to confirm and quantify the presumed effect. Third, most nerve segments available for evaluation were formalin-fixed and paraffin-embedded. While the structural detail in these specimens was less than that provided by resin-embedded semi-thin sections, unequivocal morphological evidence of nerve injury was present in the paraffin-embedded sections. Semi-thin sections of resin-embedded nerve segments, which was performed for one treated and one untreated nerve segment, demonstrated morphological evidence of injury quite consistent with that noted in the paraffin-embedded tissue [35]. Lastly, the target chosen in this pilot study was mostly the sciatic nerve to establish the feasibility of the procedure in a large nerve. Large treatment cells were used to cover the nerve in a single exposure to induce uniform ablation generating significant muscle damage in the longitudinal direction. In this pilot study, no attempts were made to reduce muscle damage as the clinical system used is not optimized for targeting nerve tissue (but rather bulk ablation of large uterine fibroids) and the primary goal was to establish the feasibility of identifying and accurately targeting nerve tissue using MR-neurography and MR-HIFU. In clinical cases, potential peripheral target nerves (e.g. posterior femoral cutaneous nerve or pudendal nerve) are smaller in diameter (e.g. 3–5 millimeters) than the porcine sciatic nerve (8–10 mm). These could be targeted with smaller treatment cells (i.e. 4 mm) and presumably higher powers and shorter sonication durations, reducing the time for heat conduction and the corresponding amount of peripheral muscle damage. This optimization of exposure parameters will be the subject of a subsequent investigation. Finally, limited thermal damage to muscle is probably tolerable in clinical application of HIFU for nerve ablation, similar to muscle damage that presumably occurs in other thermal nerve treatments such as RF ablation.

Despite these limitations, this study explores the advantages of using MR-neurography to target and evaluate peripheral nerve ablation with a clinical MR-HIFU system. The results of this study should therefore be interpreted as a proof-of-concept, serving as a basis for further technical and preclinical research for the potential application of the non-invasive treatment of peripheral neuropathy with MR-HIFU. Future studies should be aimed at the evaluation of nerve function after MR-HIFU ablation. Due to the practical purposes, a small animal model would be most suitable for such a survival study. Dedicated preclinical focused ultrasound systems for small animals are available which can be used on 3T or 7T MRI systems, depending on local availability [36–38]. Treatment cells used in small animal studies should be reduced in size to a few mm since the sciatic nerves in rodents is on the order of 1 mm. While rodents may present the most convenient model in terms of cost and size, avoiding excessive muscle ablation may be challenging. Rabbits may be a good compromise in terms of size and have been used previously for survival studies [23]. Furthermore, a survey of suitable clinical applications should be made with respect to accessibility with MR-HIFU and expected outcome.

In conclusion, the results of this pilot study indicate that 3D MR neurography-guided MR-HIFU may be a promising new technique for accurate targeted peripheral nerve ablation and provide a basis for preclinical follow-up studies.

## Supporting Information

S1 Data The supplemental table contains the sonication parameters and measured lesion characteristics for all sonications. The dimensions for contrast-enhanced T1-weighted imaging and SHINKEI imaging are provided. The dimensions of lesion measured on gross dissection as well as the dimensions of the 240 and 30 CEM43 thermal dose contours are also given.
(XLSX)
